# Machine Learning to Detect Posture and Behavior in Dairy Cows: Information from an Accelerometer on the Animal’s Left Flank

**DOI:** 10.3390/ani11102972

**Published:** 2021-10-15

**Authors:** Paolo Balasso, Giorgio Marchesini, Nicola Ughelini, Lorenzo Serva, Igino Andrighetto

**Affiliations:** Dipartimento di Medicina Animale, Produzioni e Salute, Università degli Studi di Padova, 35020 Legnaro, Italy; p.balasso@hotmail.it (P.B.); nicola.ughelini@gmail.com (N.U.); lorenzo.serva@unipd.it (L.S.); igino.andrighetto@unipd.it (I.A.)

**Keywords:** ruminant, precision livestock farming, animal welfare, triaxial accelerometer

## Abstract

**Simple Summary:**

This study analyzed the possibility of automatically detecting dairy cow behavior by combining the use of a single triaxial accelerometer applied to the animal’s left flank with a machine learning technique. This combination enabled the detection of posture and the main types of behavior that are extremely useful in evaluating the animal’s welfare and health such as resting, feeding, and rumination with a high degree of accuracy. The novelty of the study was the success in reaching a high accuracy in detecting five different behaviors and the animal posture by using a single sensor and allowing farmers to save money. To the best of our knowledge, this is the first study that has successfully explored the feasibility of locating a sensor on the animal’s left flank, showing the opportunity of automatically measuring some physiological parameters, such as those ones related to respiration and rumen health, in a non-invasive way.

**Abstract:**

The aim of the present study was to develop a model to identify posture and behavior from data collected by a triaxial accelerometer located on the left flank of dairy cows and evaluate its accuracy and precision. Twelve Italian Red-and-White lactating cows were equipped with an accelerometer and observed on average for 136 ± 29 min per cow by two trained operators as a reference. The acceleration data were grouped in time windows of 8 s overlapping by 33.0%, for a total of 35,133 rows. For each row, 32 different features were extracted and used by machine learning algorithms for the classification of posture and behavior. To build up a predictive model, the dataset was split in training and testing datasets, characterized by 75.0 and 25.0% of the observations, respectively. Four algorithms were tested: Random Forest, K Nearest Neighbors, Extreme Boosting Algorithm (XGB), and Support Vector Machine. The XGB model showed the best accuracy (0.99) and Cohen’s kappa (0.99) in predicting posture, whereas the Random Forest model had the highest overall accuracy in predicting behaviors (0.76), showing a balanced accuracy from 0.96 for resting to 0.77 for moving. Overall, very accurate detection of the posture and resting behavior were achieved.

## 1. Introduction

Livestock farming has changed over the years, increasing the number of animals per farm; this trend has drastically decreased the time that farmers and workers spend on animal observation, housing control [1], and feed management [2]. In order to make up for this lack, farmers are resorting to technology that helps them to guarantee a continuous control of many factors, including housing conditions [3], feed quality and consistency [4,5], and animal health [6,7]. With this regard, knowing the time spent by an animal standing or lying, feeding or ruminating, are of critical importance to detect the onset of a disease and assist in feeding and herd management [8,9]. Stangaferro and colleagues [7], for example, highlighted that mastitis in dairy cows is associated with changes in rumination time and physical activity, underlining that analyzing data on behavior and posture can help farmers and veterinarians in spotting changes from normality in a timely manner. This capacity allows the detection of pathologies in their onset phase [10,11], limiting production losses, preserving animal welfare [8] and contributing to ameliorate consumer perceptions of farming practice [12]. Measuring lying, standing, and ruminating bouts and localizing the main farm or pasture areas where cows behaved in such ways could also be significant information for animal management and welfare monitoring [6,11]. Among technologies applied directly on the animal, promising results have been attained with triaxial accelerometers, as reported by different authors [13,14].

Accelerometers have shown to be effective in detecting behaviors of cattle, such as ruminating, feeding, lying, or walking [15,16,17], depending on the site where the sensor is fixed to the animal. Although in previous studies a satisfactory detection of few behaviors was reached, the prediction performance tended to drop when both posture and behaviors (feeding, ruminating, walking, resting, etc.) were investigated [17,18,19]. For this reason, sometimes sensors have been used together in multiple locations [13,14], but this implies greater costs and more drawbacks for both the animal and the handler [17]. So far, using a single accelerometer to measure posture, multiple behaviors, physiological parameters, and physiological functions appear to represent a challenge. Arai and colleagues [20] applied an accelerometer into a rumen bolus to measure rumen motility in cows, but no behaviors or postures were detected, whereas Lush et al. [21] were able to detect multiple behaviors and the urination frequency in sheep. To develop this further, we thought of a position on the animal where a single sensor could give as much information as possible, both in terms of the behavior and physiological parameters. In ruminants, the left flank paralumbar fossa, beyond behavior, potentially enables the monitoring of rumen and thoraco-abdominal contractions associated with breathing and involved in both urination and defecation [22]. So far, we explored the feasibility of collecting data from this site, focusing at first on behavior detection, with the purpose of expanding the analysis to physiological data in the next future.

The aim of the present observational study was to develop a model to identify the animal posture and predefined behaviors (moving, feeding, resting, ruminating, and standing still) from data collected by a triaxial accelerometer located on the left flank paralumbar fossa of dairy cows and evaluate its accuracy and precision as well.

## 2. Materials and Methods

### 2.1. Ethical Statement

Experimental procedures were carried out in accordance with EU Directive 2010/63/EU for animal experiments and were approved by the animal welfare committee (Organismo Preposto al Benessere Animale committee—OPBA—official number 167326) of Padova University.

### 2.2. Animals, Housing and Collection Time

The trial was carried out in the Veneto region (north-eastern Italy), in a dairy farm raising cows in a loose housing facility with a concrete floor. The resting area is characterized by cubicles filled with straw and the feed bunk by the presence of headlocks. Both the number of cubicles and headlocks exceed the number of animals by around 15%. Lactating cows were fed a total mixed ration characterized by a dry matter (DM) of 48.5%, and on a DM basis by 16.0% of crude protein, 3.5% of ether extract, 34.0% of neutral detergent fiber, and 26.0% of starch. Animals were fed once a day, at 0900 h, after morning milking and milked twice a day at 0700 and 1900 h. Data were collected from 12 randomly selected Italian Red-and-White mid-lactation dairy cows, showing no signs of lameness or other diseases that might affect their behavioral repertoire; they were characterized by having on average 2.87 ± 0.91 lactations and being at 180 ± 35 days in milk. After two months spent trying the best solutions to apply the sensor to the animals and training the observers to recognize the various behaviors and postures, on April 2019, data collection started, and it was carried out on average for 136 ± 29 min per cow for 12 cows, by applying the sensor to one cow at a time (one cow a day), for 12 days on end, weekends excluded. This time was long enough to collect a sufficient amount of acceleration data from each animal during all behaviors selected to build and train the models. Animals were observed approximately between 1100 h and 1500 h, because within this interval cows were more likely to perform a variety of behaviors, including feeding, resting, ruminating, and moving (locomotion activity).

Upon the application of the sensor, each animal was observed continuously by two trained observers, who recorded animal posture and behavior in real time using Microsoft Excel 2010 (Microsoft, Remond, WA, USA) on a laptop synchronized with the triaxial accelerometer [13]. Inter-observer reliability was previously calculated using Cohens’ kappa [23] during the training period and resulted in 0.99 for posture and 0.91 for behavior.

### 2.3. Sensor Characteristics and Application, Postures and Behaviors

**Figure 1:**
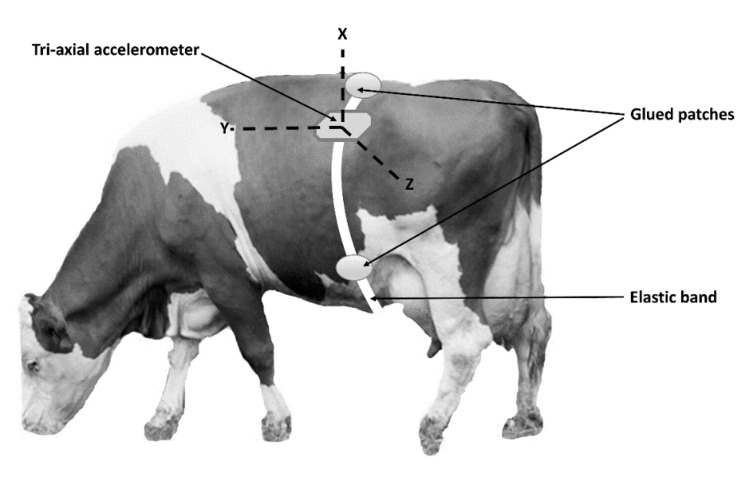
Location and position of the triaxial accelerometer on cow’s left side paralumbar fossa. The patch glued on the right side of the animal is not visible. X, Y, and Z are the acceleration axes.

The sensor used was a commercial triaxial (X, Y, Z) accelerometer equipped with data-logger, model MSR145W (PCE Italia srl, Capannori, LU, Italy), weighing 18 g, 18 mm wide, 14 mm deep, and 62 mm long, with a memory capacity of 2 million data and a detection rate ranging from 50 Hz to every 12 h. We did not associate the accelerometer to a transmitter, because at this phase, we were mainly interested in exploring the opportunity of collecting data from the animal left flank and converting them in information useful to the farmer. The sensor was set to collect data every 0.2 s (5 Hz) and was inserted in a suitable protective support to prevent the sensor from being damaged by impacts and smashing. The frequency of 5 Hz was a good compromise between the opportunity to characterize even short-term and intermittent behaviors, such as moving, which requires a high frequency and the necessity of prolonging the battery life and optimizing the data storage capacity. The support was glued to the animal’s hair and held in position by an elastic band that was in turn held in position by three patches glued to the hairs on the back and on either side of the abdomen, as reported in Figure 1. The glue used was a non-toxic mixture of natural latex, acrylate terpolymer, and ammonium hydroxide (Salon Pro, Glendale Heights, IL, USA) with a rapid drying period (from 30 s to 60 s). This product showed excellent grip on the animal’s hair. The sensor was positioned so as to have, with the animal in a standing position, the y axis parallel to the ground, the x axis in a vertical position, and the z axis orthogonal to the side of the animal, as shown in Figure 1.

The animal posture was classified as left sternal recumbency (LSR), right sternal recumbency (RSR), and standing (S); behaviors were classified as moving, standing still, feeding, ruminating, and resting. All postures and behaviors are described in Table 1.

### 2.4. Data Processing and Algorithm Description

Data were collected using the sensor software, MSR 5.12.04 (PCE Italia srl, Capannori, LU, Italy), which gave the output as a .csv file, where for each line (collection time: date, h, min, s, hundredths of a second) acceleration values on the axes X, Y, and Z were reported in different columns. This file was imported to Excel 2010 (Microsoft, Remond, WA, USA), and every change in posture or behavior recorded by observers was reported beside the acceleration values for each time interval. Statistical analyses were carried out using R computing environment, version 3.2.1 (R Core Team 2013, R Foundation for Statistical Computing, Vienna, Austria). The input dataset had for each record the tri-axial accelerations, which were collected every 0.2 s. The total number of observations from the 12 cows was 490,900, which is equal to 27.3 h, in line with other similar experiments [15,25]. The observations that the researchers were not able to assign to a univocal behavior or posture were removed from the dataset. Table 2 shows the total number of observations collected, removed, and used for the analyses of posture and behavior of each cow, showing that 1.89 h of observations were excluded, whereas the remaining 25.3 were used for the analyses.

The remaining 456,730 observations were divided into 3 postures and 5 behaviors, as shown in Table 3.

### 2.5. Feature Extraction

The magnitude of the acceleration (amag) has been estimated for each observation as the root squared of the sum of the squared tri-axis accelerations (x, y, and z): $\mathrm{amag}=\sqrt{x^{2}+y^{2}+z^{2}}$. Then, for each of the previous 4 variables (x, y, z, amag), the following functions have been applied to create a tidy dataset. A short time window of 8 s (40 observations) has been found to be the best fitting to compute a list of metrics to be used in the model. These time windows overlap by 33.0%. The elements of the feature vector consist of:

- Average (avg)
- Standard deviation (sd)
- Number of zero crossings (zc)—number of times related to a change of sign (after scaling it).
- Peak-to-peak value (p2p)—difference between the highest and the lowest value within the interval.
- Root mean squared value (rms)
- Kurtosis (kur)
- Skewness (skw)
- Crest factor (cf)—the ratio between the maximum peak and the root mean squared value.
- RMS of the integral (RMS of the velocity, Vrms)—this can be obtained by computing the inverse function of the lagged differences function of a vector and calculating the root mean squared value.

Each interval of 40 observations with a sliding interval of 13 observations (33.0% of 40 observations) has been chosen as the observation unit. Hence, a dataset of 35,133 rows (456730/13) was obtained. However, all the intervals that contained more than one consecutive behavior or posture were excluded from the analysis. Two different datasets were created: one to predict the behavior and the other to determine the posture. Overall, the behavior dataset included 33,318 observations and the posture dataset 32,500. The variables recorded for each 8 s time interval are reported in Table 4.

Variables that were highly correlated and showed a cut-off value equal or higher to 0.8, were removed: amag.rms, z.sd, z.p2p, amag.p2p, amag.cf, amag.sd, x.p2p, amag.avg, x.rms, y.sd, x.Vrms, x.avg, z.avg, z.Vrms, y.Vrms, y.zc, y.kur, y.skw. To build up a predictive model, the dataset was randomly split in a training (75.0% of the observations) and testing (25.0%) datasets. The latter has been used to estimate the performance of the model. All variables have been normalized considering the mean and the standard deviation of the training dataset. For posture, two datasets of 24,377 and 8123 observations were attained for training and testing, respectively, whereas for behavior, the training and testing datasets included 22,701 and 7567 observations.

### 2.6. Data Modelling

To find the most suitable algorithm to predict both posture and behavior, 4 different algorithms were run: the Support Vector Machine (SVM), Random Forest (RF), K Nearest Neighbors (KNN), and Extreme Boosting Algorithm (XGB). All of them have already proved to be useful in classifying behaviors in cows as reported in the literature [11,17]. The R packages used to apply the different algorithms are described in [26] for SVM, [27] for RF, [28] for KNN, and [29] for XGB.

### 2.7. Model Assessment

To compare the overall ability of different models in predicting either the animal posture or behavior, Cohen’s kappa (K), accuracy, accuracy lower, accuracy upper, and accuracy *p*-value were reckoned [30]. The upper and lower accuracy are calculated considering a confidence interval of 95.0%. The confidence interval for the accuracy rate uses the default binomial confidence interval method used in the binon.test function [31] in R. Cohen’s kappa gives an estimate of agreement between the algorithm and the observation results, corrected for hypothetical probability of chance agreement [23], whereas accuracy, once, for each model, the number of true positive (TP), true negative (TN), false positive (FP) and false negative (FN) has been established, is reckoned as accuracy = (TP + TN)/(TP + FP + FN + TN), and gives an overall measure of correctly identified postures or behaviors [15]. For each model, the sensitivity, specificity, positive predictive value (PPV or precision), and negative predictive value (NPV) were calculated to determine each posture or behavior. These parameters were calculated as follows: sensitivity = TP/(TP + FN), specificity = TN/(TN + FP), PPV or precision = TP/(TP + FP), and NPV = TN/(TN + FN), as reported in the literature [15,32]. In addition, for each posture or behavior, its prevalence was given, reckoned as the ratio between the number of its occurrence and the number of all postures or behaviors recorded, and its balanced accuracy, calculated as (sensitivity + specificity)/2.

## 3. Results

### 3.1. Posture Model Results

The accuracy related to the posture for each model is reported in Table 5. It appears that the model with the best performance is XGB with an accuracy of about 99.2% and the highest Cohen’s kappa.

As described in Table 6, the prediction of posture was found to be accurate for all the models, always being higher than 0.940. Moreover, all the models displayed a high range of sensitivity (0.89–1.00), specificity (0.961–0.999), precision (0.927–0.996), and NPV (0.981–1.00). The highest prevalence was detected for standing, whereas the lowest was for LSR.

Table 7 shows the confusion matrix for each model applied to the testing dataset for the determination of posture. Although overall all the models are reported to predict the posture very well, it is worthwhile noticing that even in the best model, XGB, there are few observations (2.49%) belonging to LSR that are predicted as S and vice versa (0.60%).

### 3.2. Behavior Model Results

With regard to the accuracy of different models in predicting animal behavior, the accuracy of different models has a range between 0.676 and 0.759, where the lowest accuracy and Cohen’s kappa are given by SVM and the highest by RF model (Table 8).

As reported in Table 9, the behavior predicted with the highest balanced accuracy is resting (0.927–0.962), followed by feeding (0.729–0.803), standing still (0.716–0.800), ruminating (0.735–0.805), and moving (0.689–0.774). The RF model led to the highest balanced accuracy, sensitivity, precision, and NPV in the prediction of feeding, moving, and standing still, whereas for the prediction of resting and ruminating the highest balanced accuracy, sensitivity, and NPV were achieved using model XGB. Overall, KNN and SVM proved to be less effective in predicting the considered behaviors compared with RF and XGB.

Table 10 shows the confusion matrix for each model applied to the testing dataset for the prediction of behavior. Even in RF, the most accurate model, behaviors are in some cases misclassified: the feeding behavior is predicted as standing still (21.7%) or moving (9.45%), moving behavior is predicted as feeding (17.7%) or standing still (21.3%), resting behavior is predicted as ruminating (2.60%), and standing still is misclassified as eating (16.0%) or moving (10.8%). The other models show generally worse predictions.

## 4. Discussion

In this research, we investigated whether data attained through a triaxial accelerometer located on the left flank paralumbar fossa of dairy cows could be useful in predicting animal’s posture and behavior. Moreover, we tried to determine what algorithm could maximize accuracy and precision of these predictions. In both the prediction of posture and behavior, the acceleration parameters that had helped the most in discriminating between different classes were related to the y and z axes, which, in our case, gave us the main information about acceleration and movements forward and backward (y), orthogonal to the animal side (z), and about body rotation on the longitudinal body axis (x and z). The prediction of posture was very accurate and precise for all the models, but particularly for XGB, and proved to be much more accurate, sensitive, and precise than that found in studies where the accelerometer was positioned on a collar or on the head. Martiskainen et al. [18] and Vázquez Diosdado et al. [25], who mounted the accelerometer on a collar, found a sensitivity of 0.80 and 0.77, respectively, and a precision of 0.80 and 0.98. A better but still less sensitive (0.94) and precise (0.96) result was attained by Roland et al. [15] in calves equipped with an accelerometer on an ear tag. The lower ability in predicting the animal posture by these studies could be partly due to the different performance of the algorithms used, and perhaps because, when lying in sternal recumbency, cattle keep their head up, in the same position of when they stand up, and the neck generally moves slightly during both standing and lying [14]. In this position, the gravity acceleration measured by the accelerometer has the same direction when the animal is standing or lying. A prediction of posture in line with our results was also found by other authors [14,33] when they applied the accelerometer to the right hind leg. When in fact the accelerometer is applied on both the paralumbar fossa or a leg, the change of posture of the animal (from standing to lying and vice versa) causes a variation in the tri-axis acceleration measured by the accelerometer on the three axes X, Y, and Z. This location of the accelerometer also allowed to distinguish between left and right sternal recumbency. For all the models, RSR resulted as the posture with the best prediction, while LSR was the one with the lowest accuracy, confirmed by a higher number of misclassifications between LSR and S. A possible explanation is that the difference of the angle between the accelerometer and the ground plane between LSR and S is lower than that between S and RSR. 

In this study, cows spent almost 60.0% of their time standing and 40.0% lying. This could be an important factor affecting the prediction reliability [17,19], but this proportion is not representative of the daily time budget, which was not the purpose of this study, because the observations were made on purpose between late morning and early afternoon, so to see a greater variety of behaviors. Being able to detect the amount of time spent by cows lying or standing is very important because it’s known that lying deprivation, by even relatively short periods, below 10.3–12.0 h/day in lactating dairy cows in confinement-housed freestall facilities, can lead to detrimental effect on health and productivity [34]. On the other hand, it is also known that lying time is negatively associated with milk yield at the individual-cow level, since high-producing animals need more time to eat and being milked [34]. The high accuracy with which the accelerometer on the left paralumbar fossa can detect the animal’s posture would allow to detect in individual cows even slight changes in the time spent lying, which if prolonged over time, could be associated with warning signals for the farmer, as reported by Stangaferro et al. [7] in the case of mastitis detection. Animals spending an inadequate time lying or experiencing sudden changes in it might be affected by cow-related factors such as parity, stage of lactation, ill health, and lameness, as reported by King et al. [35], and the farmer has the opportunity to focus his attention on the reported animal and discriminate among different causes. In case inadequate lying time or its changes over time affect a group of animals in the same period, the cause it is more likely linked to housing or management issues [34]. The latter include the design and surface of the resting area, overstocking, thermal conditions, time spent outside the stall for milking, etc. [34].

Compared with posture, the prediction of behavior was less performing, given that even RF, which indeed was the best model, showed an overall accuracy in the prediction of behavior of 0.759. This is likely due partly because we tried to differentiate a good number of different behaviors, and because some of these behaviors, such as feeding and ruminating, were characterized more by movements of the head and less by movements and position of the body. Our outcomes were very good in predicting resting behavior (accuracy of 0.96) and were in line with other studies that tried to predict multiple behaviors with a single sensor, such as Roland et al. [15], who found an overall accuracy of 0.708, and Martiskainen et al. [18], who reported, for example, a slightly lower precision in predicting rumination (0.785) but higher sensitivity and precision for feeding (0.75 and 0.81) and walking (0.79 and 0.79). Other authors, who applied the accelerometer to the collar and limited the number of behaviors investigated, found for feeding a sensitivity ranging from 0.93 to 0.98 [17,25], whereas, combining behavior and posture (e.g., ruminating–standing or ruminating–lying), other researchers found a sensitivity and specificity up to 0.97 and 0.99, respectively [17].

In the present study, for all the predictive models, the most common misclassifications were about behaviors classified as standing still or feeding instead of moving. This probably depends on the fact that cows moving slightly during exploration or grooming activities, making few steps in a minute, can be easily confused with those standing still or feeding, as reported by Benaissa et al. [14], when using leg accelerometers. Resting was commonly mistaken for ruminating and feeding was commonly misclassified for moving. Again, resting and ruminating behaviors, especially when lying, have similar movement patterns, differing almost only for the movement of the jaws and the head.

All the algorithms tested in this study to build the best fitting models have already been used in this field for some years such as SVM and KNN [14,18], and the ones which allowed us to build the best fitting models, such as RF and XGB, are gaining more and more importance for the diagnosis of diseases [11] and the detection of behaviors, and are reported to give better predictions than the others, as found in our study [17]. Both of them are effective algorithms and present elements that allow high performance. The RF is less likely to overfit by averaging several trees, but at the same time, it has low variance: by using multiple trees, it reduces the chance of stumbling across a classifier that does not perform well because of the relationship between the training and testing data. The XGB is another algorithm that can perform implicit variable selection, can capture non-linear relationships in the data, high-order interactions between inputs, and scale well to large datasets. Comparing these two models, if parameters are carefully tuned, XGB can result in better performance than RF. However, XGB may not be a good choice if there is a lot of noise, as it can result in overfitting. XGB also tends to be harder to tune up than RF. Behaviors such as ruminating and feeding are extremely important to assess whether the chemical and physical characteristics of the ration are adequate but may also be affected by housing and management strategies, such as the amount of space at the feed bunk, the frequency with which feed is pushed-up in the bunk, etc. [36]. Furthermore, variation in the activity and rumination pattern are reported to be extremely useful in the early detection of disease onset [7,10]. Since, so far, the accuracy of rumination detection with our method is around 0.80, it appears to be more suitable to detect rumination changes in a group, due to mistakes in diet preparation or management, than in individuals, due to the onset of a disease. Overall, our results refer to mid-lactating cows belonging to a single breed, housed in one farm and sampled for one day. Although they look promising, their application to commercial farms requires first their confirmation from further studies taking into account different breeds and housing and environmental conditions. Furthermore, during 24/7 monitoring, because of the impossibility of removing transition behaviors, we could experience a slightly lower accuracy for short-term behaviors such as moving and standing still. As regards the position of the sensor on the left flank, the harness used in this experiment was tested during the pre-trial period and allowed to keep the sensor in position for up to 50 days. The elastic band helped to prevent the other cows from pulling off the accelerometer. Despite the harness used working quite well during the experiment, the application method of the sensor should be simplified and improved. Moreover, further trials should be carried out by connecting the accelerometer to a transmitter to allow the continuous collection of data remotely.

## 5. Conclusions

Overall, the application of a single triaxial accelerometer at the left side paralumbar fossa of mid-lactating dairy cows has given very accurate results concerning the prediction of posture and the resting behavior, whereas other behaviors such as standing still, feeding, moving, and ruminating overall showed an accuracy below 0.80, which is lower than that found in other studies. These outcomes demonstrate that this location has a great potential for the collection of important data in dairy cows. Further studies on the improvement of the application method and the feasibility of predicting physiological data from the same sensor in the same position are warranted.

## Figures and Tables

**Table 1:** Posture and behavior description for dairy cattle.

	Posture ^1^ or Behavior ^2^	Definition ^3^
Posture	Standing	Cows stand at three or four legs
	Right sternal recumbency	Cows lie on the sternum with the hind legs on the left
	Left sternal recumbency	Cows lie on the sternum with the hind legs on the right
Behavior	Standing still	Cows are in a standing position and do not move their legs or show any sign of activity
	Feeding	Cows actively intake the feed at the manger including chewing it in the manger space
	Moving (Walking or moving slightly)	Cows in standing position walk through the pen or perform any behavior, different from those mentioned above, which includes at least a step every 10 s.
	Ruminating	Cows perform chewing movements; it begins when the cow starts to chew a regurgitated bolus and it ends when the bolus is swallowed back. It can be done both while the animal is standing or lying.
	Resting	Cows lie, not moving or ruminating

^1^ Animal posture was categorized as: standing, right sternal recumbency and left sternal recumbency. ^2^ Animal behavior was categorized as: standing still, feeding, moving (walking or moving slightly), ruminating and resting. ^3^ Adapted from Cortese et al. [24].

**Table 2:** Distribution of observation data among cows pre- and post-removal of observations not referring to univocal behavior or posture.

Cow	Total Number of Observations	Number of Removed Observations	Number of Observations Used	Minutes of Removed Observations	Minutes of Observation Used
1	59,628	1892	57,736	6.3	192
2	45,927	3902	42,025	13	140
3	34,468	619	33,849	2.1	113
4	30,263	582	29,681	1.9	99
5	394,05	7794	31,611	26	105
6	31,758	3201	28,557	10.7	95
7	40,784	1200	39,584	4	132
8	37,047	2527	34,520	8.4	115
9	45,039	1680	43,359	5.6	145
10	36,894	1350	35,544	4.5	118
11	52,770	7759	45,011	25.9	150
12	36,917	1664	35,253	5.5	118
Total	490,900	34,170	456,730	113.9	1522

**Table 3:** Observation time spent by cows in different postures and behaviors.

Posture	Total Observations	Hours of Observations
Left sternal recumbency	67,771	3.76
Right sternal recumbency	115,736	6.43
Standing	273,222	15.2
**Behavior**		
Feeding	84,206	4.68
Moving	84,400	4.69
Resting	141,055	7.84
Ruminating	53,744	2.98
Standing still	93,325	5.18

**Table 4:** Variables obtained for each 8 s time window concerning axes x, y, z, and the magnitude of acceleration (amag).

X	Y	Z	Amag
x.avg	y.avg	z.avg	amag.avg
x.sd	y.sd	z.sd	amag.sd
x.zc	y.zc	z.zc	amag.zc
x.p2p	y.p2p	z.p2p	amag.p2p
x.rms	y.rms	z.rms	amag.rms
x.kur	y.kur	z.kur	amag.kur
x.skw	y.skw	z.skw	amag.skw
x.cf	y.cf	z.cf	amag.cf
x.Vrms	y.Vrms	z.Vrms	amag.Vrms

Avg = average; sd = standard deviation; zc = number of zero crossing; p2p = peak to peak value; rms = root mean squared value; kur = kurtosis; skw = skewness; cf = crest factor; Vrms = RMS of the velocity.

**Table 5:** Model accuracy for the prediction of posture of the testing dataset.

Model	Accuracy	Cohen’s Kappa	Accuracy Lower	Accuracy Upper	Accuracy *p*-Value
RF	0.988	0.978	0.985	0.990	<0.001
KNN	0.974	0.954	0.971	0.978	<0.001
XGB	0.992	0.986	0.990	0.994	<0.001
SVM	0.976	0.957	0.973	0.980	<0.001

RF = Random Forest model; KNN = K Nearest Neighbors model; XGB = Extreme Gradient Boosting model; SVM = Support Vector Machine model.

**Table 6:** Model sensitivity, specificity, precision, negative predictive value, prevalence, and accuracy for the prediction of posture.

Posture	Model	Sensitivity	Specificity	Precision	NPV	Prevalence	Balanced Accuracy
RSR	RF	1.00	0.999	0.996	1.00	0.253	0.999
LSR	RF	0.946	0.996	0.973	0.991	0.148	0.971
S	RF	0.993	0.982	0.988	0.989	0.599	0.987
RSR	KNN	0.994	0.995	0.987	0.998	0.253	0.995
LSR	KNN	0.932	0.987	0.927	0.988	0.148	0.960
S	KNN	0.977	0.972	0.981	0.966	0.599	0.974
RSR	XGB	1.00	0.999	0.998	1.00	0.253	1.00
LSR	XGB	0.971	0.996	0.976	0.995	0.148	0.983
S	XGB	0.994	0.991	0.994	0.991	0.599	0.992
RSR	SVM	0.998	0.999	0.996	0.999	0.253	0.998
LSR	SVM	0.890	0.992	0.951	0.981	0.148	0.941
S	SVM	0.989	0.961	0.974	0.983	0.599	0.975

Model = model of the algorithm used for data analysis; NPV = negative predictive value; RSR = right sternal recumbency; LSR = left sternal recumbency; S = standing; RF = Random Forest model; KNN = K Nearest Neighbors model; XGB = Extreme Gradient Boosting model; SVM = Support Vector Machine model.

**Table 7:** Model confusion matrix for the prediction of posture.

Model	Predicted	Actual			
		RSR	LSR	S	Total
RF	RSR	2058	6	3	2067
RF	LSR	0	1137	31	1168
RF	S	1	59	4828	4888
KNN	RSR	2047	3	25	2075
KNN	LSR	0	1120	88	1208
KNN	S	12	79	4749	4840
XGB	RSR	2059	5	0	2064
XGB	LSR	0	1167	29	1196
XGB	S	0	30	4833	4863
SVM	RSR	2055	9	0	2064
SVM	LSR	0	1070	55	1125
SVM	S	4	123	4807	4934
Total		2059	1202	4862	

Model = model of the algorithm used for data analysis; PPV = positive predictive value; NPV = negative predictive value; RSR = right sternal recumbency; LSR = left sternal recumbency; S = standing; RF = Random Forest model; KNN = K Nearest Neighbors model; XGB = Extreme Gradient Boosting model; SVM = Support Vector Machine model.

**Table 8:** Model accuracy for the prediction of behavior.

Model	Accuracy	Cohen’s Kappa	Accuracy Lower	Accuracy Upper	Accuracy *p*-Value
RF	0.759	0.687	0.749	0.768	<0.001
KNN	0.681	0.587	0.670	0.692	<0.001
XGB	0.736	0.657	0.726	0.746	<0.001
SVM	0.676	0.578	0.665	0.687	<0.001

Model: model of the algorithm used; RF = Random Forest model; KNN = K Nearest Neighbors model; XGB = Extreme Gradient Boosting model; SVM = Support Vector Machine model.

**Table 9:** Model sensitivity, specificity, precision, negative predictive value, prevalence, and accuracy for the prediction of behavior.

Behavior	Model	Sensitivity	Specificity	Precision	NPV	Prevalence	Balanced Accuracy
Feeding	RF	0.685	0.922	0.671	0.926	0.189	0.803
Moving	RF	0.597	0.951	0.706	0.923	0.164	0.774
Resting	RF	0.964	0.956	0.915	0.982	0.331	0.960
Ruminating	RF	0.601	0.987	0.873	0.945	0.125	0.794
Stand still	RF	0.718	0.881	0.588	0.930	0.191	0.800
Feeding	KNN	0.587	0.902	0.583	0.904	0.189	0.745
Moving	KNN	0.518	0.926	0.579	0.907	0.164	0.722
Resting	KNN	0.918	0.936	0.877	0.959	0.331	0.927
Ruminating	KNN	0.618	0.950	0.641	0.946	0.125	0.784
Stand still	KNN	0.545	0.887	0.531	0.892	0.191	0.716
Feeding	XGB	0.627	0.917	0.638	0.914	0.189	0.772
Moving	XGB	0.563	0.945	0.666	0.917	0.164	0.754
Resting	XGB	0.964	0.960	0.922	0.982	0.331	0.962
Ruminating	XGB	0.627	0.982	0.834	0.948	0.125	0.805
Stand still	XGB	0.669	0.866	0.542	0.917	0.191	0.768
Feeding	SVM	0.549	0.910	0.587	0.897	0.189	0.729
Moving	SVM	0.415	0.962	0.681	0.893	0.164	0.689
Resting	SVM	0.933	0.924	0.858	0.965	0.331	0.928
Ruminating	SVM	0.487	0.983	0.809	0.930	0.125	0.735
Stand still	SVM	0.705	0.810	0.468	0.921	0.191	0.758

Model = model of the algorithm used for data analysis; NPV = negative predictive value; RF = Random Forest model; KNN = K Nearest Neighbors model; XGB = Extreme Gradient Boosting model; SVM = Support Vector Machine model.

**Table 10:** Model confusion matrix for the prediction of behavior.

Model	Predicted	Actual					
		Feeding	Moving	Resting	Ruminating	Standing still	Total
RF	Feeding	978	220	8	20	232	1458
RF	Moving	135	742	3	15	156	1051
RF	Resting	4	9	2412	204	8	2637
RF	Ruminating	1	6	65	570	11	653
RF	Standing still	310	265	13	140	1038	1766
KNN	Feeding	839	251	18	34	297	1439
KNN	Moving	202	643	18	29	218	1110
KNN	Resting	28	24	2296	219	52	2619
KNN	Ruminating	48	40	150	587	91	916
KNN	Standing still	311	284	19	80	787	1481
XGB	Feeding	896	202	5	21	280	1404
XGB	Moving	161	699	7	11	171	1049
XGB	Resting	3	13	2410	182	6	2614
XGB	Ruminating	8	20	69	595	21	713
XGB	Standing still	360	308	10	140	967	1785
SVM	Feeding	784	272	16	18	246	1336
SVM	Moving	115	516	10	7	110	758
SVM	Resting	14	28	2334	286	59	2721
SVM	Ruminating	1	15	82	462	11	571
SVM	Standing Still	514	411	59	176	1019	2179
Total		1428	1242	2501	949	1445	

Model = model of the algorithm used for data analysis; RF = Random Forest model; KNN = K Nearest Neighbors model; XGB = Extreme Gradient Boosting model; SVM = Support Vector Machine model.

## Data Availability

The data presented in this study are available on request from the corresponding author.
